# Lumbar Spine Fusion Rates With Local Bone in Posterolateral and Combined Posterolateral and Interbody Approaches

**DOI:** 10.5435/JAAOSGlobal-D-18-00018

**Published:** 2019-11-04

**Authors:** Daniel K. Park, Richard Roberts, Paul Arnold, David H. Kim, Rick Sasso, Kevin C. Baker, Jeffrey S. Fischgrund

**Affiliations:** From the Department of Orthopaedic Surgery, Beaumont Health, Royal Oak, MI (Dr. Park, Dr. Roberts, Dr. Baker, and Dr. Fischgrund); the Department of Neurosurgery, University of Kansas Hospital, Kansas City, KS (Dr. Arnold); the Department of Orthopaedic Surgery, New England Baptist Hospital, Boston, MA (Dr. Kim); and Indiana Spine Center, Caramel, IN (Dr. Sasso).

## Abstract

Posterolateral lumbar fusion (PLF) used to treat degenerative lumbar conditions still faces pseudarthrosis. Bone graft choice is a key factor; a traditional choice has been autologous iliac crest bone graft (ICBG), but complication rates are quoted up to 39%. Local bone from laminectomy eliminates ICBG harvesting complications.

**Methods:**

Two hundred forty-one patients underwent either PLF or PLF with interbody at a single lumbar level with a prospective, multicenter, randomized controlled trial only using local bone graft. Fusion was assessed with radiographs and CT.

**Results:**

PLF fused bilaterally in 18% and unilaterally in 28.8% at 6 months and 35.7% and 50.3% at 12 months, respectively. At 6-month PLF + interbody, 1.1% fused bilaterally and 11.7% unilaterally; at 12 months, 5.4% fused all three areas, and 50.8% fused at least one area.

**Discussion:**

Local bone fused substantially less than the “benchmark” ICBG.

Posterolateral lumbar fusion (PLF) is the most common method surgeons use to treat degenerative lumbar conditions; yet, pseudarthrosis still occurs. Many factors determine success of fusion, particularly the surgeon's choice of bone graft. Autologous iliac crest bone graft (ICBG) has been considered the “benchmark” for fusion^1-3^; yet, complications with harvesting have been extensively reported.^4,5,6,7,8^ Kurz et al^7^ reported a major complication rate of 10% while minor complications were 39%. Even minor complications can lead to disability, increased recovery time, and patient care costs.

Because of the morbidity of ICBG harvesting, potential alternatives have been pursued, one being the local bone harvested from the decompression, a collection of large amount of autogenous posterior element bone. Inage et al^9^ found in 1- or 2-level fusions, local bone alone sufficed in producing an acceptable fusion rate; however, three-level fusions faired poorly with a fusion rate of 62.5%. Other studies have also analyzed fusion rates of local bone use only; yet, they were small in number and single-center studies.^9,10,11,12,13,14^

To augment fusions, many surgeons use an interbody (IB) technique which provides another fusion area and anterior column support. Good clinical outcomes and fusion rates have been reported.^13,14^ However, as with using local bone alone, the studies are typically reported on a small number of patients. A larger, multicenter study is needed to assess whether local bone alone in the setting of instrumented PLF with or without IB support leads to an adequate fusion rate. The goal of this study was to evaluate the fusion rate of a prospectively gathered multicenter cohort undergoing laminectomy with a single-level instrumented fusion with or without IB support.

## Methods

### Study Design

A multicenter, prospective, double-blind, randomized, placebo-controlled pivotal study was undertaken to examine the adjunctive effects of ultrasonography therapy in the promotion of arthrodesis in single-level posterior, instrumented lumbar spine fusion. The study was conducted in accordance with a protocol approved by the institutional review boards of each of the participating sites and was also posted on clinicaltrials.gov (NCT00744861—Ultrasound as Adjunct therapy for Increasing Fusion Success after Lumbar Surgery). The study included 26 sites throughout the United States and enrolled 310 patients before discontinuation. All patients underwent an instrumented PLF with only autologous local bone graft. No statistically significant differences (*P* = 0.9905) were identified between the ultrasound-treated and control subjects in fusion status or clinical outcome measures. As such, the prospectively collected data were retrospectively reviewed to characterize the efficacy of autologous local bone graft in the promotion of posterolateral and/or IB fusion at a single instrumented lumbar level.

### Surgical Procedure

Patients aged 18 to 81 years with evidence of single-level degenerative disk disease, with or without spondylolisthesis (maximum grade I), who failed at least six months of nonsurgical treatment were enrolled in the study. In addition, the patients demonstrated one or more of the following findings: radiographic evidence of instability; facet joint or end plate osteophytes; disk space narrowing; scarring or thickening of the facet joint capsule, annulus fibrosis or ligamentum flavum; herniated nucleus pulposus; facet joint degeneration; and/or vacuum phenomenon. After consent and collection of baseline data, patients underwent a surgical decompression of the level to be fused, with additional decompression at other levels permitted. After adequate decompression, single-level fusion was done using titanium pedicle screws and 5.5-mm-diameter metallic rods as dictated by FDA indications. In addition to PLF, some patients also underwent a concomitant IB fusion at the same level facilitated by placement of a rectangular IB cage composed of titanium, polyetheretherketone, or carbon-fiber-reinforced polyetheretherketone. Autologous local bone harvested during the decompression was placed in the posterolateral gutters bilaterally bridging the intertransverse process space, as well as packing the IB cage; no additional graft material, graft extenders, or enhancers were used. If the surgeon felt the amount of bone graft was inadequate for fusion, the patient was excluded from the study. An absolute cutoff of bone graft volume was not established. Patients followed up with their attending surgeon at 2- and 6-week, 3-, 6-, 12-, and 24-month intervals postoperatively.

### Characterization of Fusion Status

To determine the fusion status, flexion-extension radiographs and a CT scan were obtained at 12 months postoperatively. Two blinded fellowship-trained musculoskeletal radiologists reviewed radiographs and CT scans. In the event of a disagreement, a third, blinded fellowship-trained musculoskeletal radiologist was used as a tiebreaker. Three criteria were used to determine the success of fusion at the surgical level:

(1) Less than 5° of angulation on flexion-extension radiographs.
(2) Less than 3 mm of translation on flexion-extension radiographs.
(3) Bridging bone connecting the transverse process on both sides on CT scans for PLF.
(4) Bridging bone with uniform radiodensity obscuring at least one end plate (IB) by new bone if posterolateral fusion with IB (PLF + IB). It should be noted for patients with PLF + IB, the first three criteria had to be met as well; thus, a solid bilateral PLF needed to be present.

CT evidence of bridging bone in patients who underwent PLFs (without IB) was graded according to the method presented by Glassman et al.^15^ A modification of this grading method was used to assess CT evidence of bridging bone in patients who underwent IB fusion along with PLF (Table 1).

**Table 1:** Characterization of CT Evidence of Bridging Bone in Patients Who Underwent Either Isolated Posterolateral or Posterolateral With IB Fusion Approaches

Surgical Approach	Grade	Description
Posterolateral	Grade 1	No fusion.
	Grade 2	Partial or limited unilateral fusion.
	Grade 3	Partial or limited bilateral fusion.
	Grade 4	Solid unilateral fusion.
	Grade 5 (fused)	Solid bilateral fusion.
Posterolateral + IB	Grade 1	Trace radiodense material at the surgical level.
	Grade 2	Flocculent radiodensity with flecks of calcification and incomplete bridging of the surgical level.
	Grade 3	Bridging of bone in at least one location with material of nonuniform radiodensity.
	Grade 4 (fused)	Bridging of bone at the surgical level with material of uniform density, obscuring of at least one end plate by new bone.
	Grade 5 (fused)	Bridging of involved vertebral end plates with new bone of uniform density.

IB = interbody

### Outcome Measures

In addition to assessing fusion on radiographs and CT, clinical assessments were done at the preoperative and postoperative visits. Neurologic status was assessed, including characterization of reflexes, sensory and motor parameters, and a straight leg raise. The intensity and frequency of back and leg pain was rated by the patients using a 10-point scale, with the total leg pain score calculated as the sum of both the intensity and frequency of pain. The Oswestry Disability Index (ODI) was administered to assess the patient's pain and disability stemming from their spinal condition.

## Results

The primary outcome of this original trial was to compare the effect of ultrasonography on fusion. An interim analysis was done on the first 159 patients who reached the 12-month time point with complete fusion assessments. At this time point, successful fusion was 32.9% in those undergoing ultrasonography treatment and 33.8% in the control subjects. The difference between the primary outcome variable (fusion status) groups was not statistically significant (*P* = 0.9905), and the study was terminated. With a large cohort of strictly single-level posterolateral fusion procedures at multiple centers that shared the same criteria, we decided to reanalyze the fusion results. Furthermore, no serious adverse events were identified as related to treatment with ultrasonography.

### Demographics

In this study, 310 patients were enrolled, 125 men (40.3%) and 185 women (59.7%) with an average age of 57.2 years (age range 22 to 81 years). Of the 310 patients, four patients underwent a fusion of L2-L3, 32 patients at L3-L4, 190 patients at L4-L5, and 84 patients underwent fusion at L5-S1. At 6 months, 231 patients had clinical and radiographic data, while only 182 patients had completed follow-up at 12 months. Of these 231 patients at 6 months, 100 patients underwent single-level laminectomy with PLF, 79 patients had single-level laminectomy with PLF + IB, 37 patients had multilevel laminectomy with PLF, and 15 had multilevel laminectomy with PLF + IB. At 12 months, 81 patients underwent single-level laminectomy with PLF, 62 patients had single-level laminectomy with PLF + IB, 27 patients had multilevel laminectomy with PLF, and 12 had multilevel laminectomy with PLF + IB. Seventy-three percent of the PLF cohort had single-level decompression at 6 months increasing to 76% at 12 months. For the PLF + IB cohort, 84% had single-level decompression with PLF + IB at 6 and 12 months.

Bone graft volume was available for 84.2% of patients at the 6-month follow-up and 82.7% of patients at the 12-month follow-up. For a single-level decompression, the mean bone graft volume was 26.7 ± 13.4 mL for PLF and 29.7 ± 14.1 mL for PLF + IB (*P* = 0.174). For multilevel decompression, the mean bone graft volume was 33.7 ± 11.5 mL for PLF and 27.6 ± 10.0 mL for PLF + IB (0.092) (Table 2).

**Table 2:** Comparison of Bone Graft Volumes Used in PLF and PLF + IF Procedures as a Function of Single- Versus Multilevel Decompressions

	6 Months		12 Months	
Decompression	PLF (mL)	PLF + IF (mL)	PLF (mL)	PLF + IF (mL)
Single-level	26.7 ± 13.4	29.7 ± 14.1	26.3 ± 13.3	29.6 ± 14.1
Multilevel	33.7 ± 11.5	27.6 ± 10.0	33.1 ± 10.4	29.2 ± 9.7

IF = interbody fusion; PLF = posterolateral lumbar fusion

Data presented as mean ± SD.

### Fusion Data

**Figure 1:**
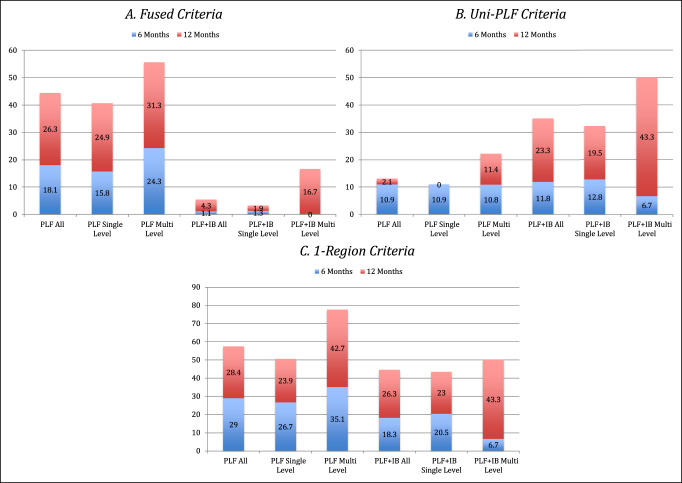
Fusion rates at 6 months and 12 months for patients undergoing PLF versus PLF + IB. Decompression categorized at all, single-level, or multilevel. Results are expressed as a function of three different criteria. **A**, Fused criteria. **B**, Uni-PLF criteria. **C**, 1-Region criteria. IB = interbody, PLF = posterolateral lumbar fusion

At 6 months, 18.1% of patients who underwent PLF were fused based on above criteria, increasing to 44.4% at 12 months; requiring only unilateral fusion instead of bilateral fusion, rates increased to 29.0% and 57.4%, respectively. When analyzing patients who underwent a single-level laminectomy, the fusion rate at 6 months was 15.8% and 40.7% at 12 months, with unilateral fusion criteria, 26.7% and 50.6%, respectively. Patients who underwent multilevel laminectomies demonstrated bilateral fusion rates of 24.3% and 55.6%, at 6 and 12 months, with unilateral fusion criteria, 35.1% and 77.8%, respectively (Figure 1).

With the inclusion of IB cage, the IB fusion was 1.1% at 6 months and 5.4% at 12 months, with the less stringent criteria, fusion of 18.3% and 44.6%, respectively. Single-level decompression with a PLF + IB had a fusion rate of 1.3% at 6 months and 3.2% at 12 months, with lenient criteria, 20.5% and 43.5%, respectively. Multilevel decompression and IB fusion had rates of 0% at 6 months and 16.7% at 12 months, with the lenient criteria rates of 6.7% and 50%, respectively. Fused patients tended to have a larger volume of bone graft, particularly those patients who were fused at 12 months (fused = 31.4 mL, not fused = 27.7 mL; *P* = 0.132).

### Clinical Score

Patients had a statistically notable improvement in ODI and visual analog score (VAS) back and leg pain scores (Table 3). However, there were no statistically notable findings between the 6- and 12-month follow-up for those who had multilevel or single-level decompression.

**Table 3:** Aggregate Outcome Scores

Factor	ODI	VAS—Back Pain	VAS—Leg Pain
Baseline	52.5 ± 13.9	15.5 ± 3.9	15.1 ± 4.1
6 months	21.9 ± 17.4	6.34 ± 5.4	5.4 ± 6.0
12 months	19.5 ± 18.2	6.0 ± 5.8	5.4 ± 6.3

ODI = Oswestry Disability Index

## Discussion

The use of local bone graft harvested during decompression for fusion has the potential to eliminate the morbidity of ICBG. The cohort was predominately a single-level decompression with an IB fusion that only used local bone gathered from the decompression. In evaluating the success of local bone graft and ultrasonography effect on fusion, no bone graft enhancers or extenders were allowed. In this study, we found local bone alone provides a low fusion rate, substantially less than the historic fusion average rates of 75% using ICBG.^10,11,16-18^ Adding more biomechanical stability with an IB fusion did not improve the fusion rate.

In comparison with other studies, our fusion rates using local bone alone was substantially less. Sengupta et al found a 65% fusion rate in their 40 cases, only using dynamic radiographs to assess fusion. In particular for single-level fusion, 24 of 30 patients fused,^11^ which substantially decreased with two or more levels of fusion. This study also allowed for multilevel decompressions. Ohtori et al^10^ compared 42 patients undergoing one-level fusion with local bone versus 40 patients fused with ICBG. The local bone cohort had a fusion rate of 83% on radiograph, compared with 85% in the ICBG cohort through CT and dynamic radiographs. Using CT, the average rate and average duration of fusion were 90% and 8.5 months for local bone and 85% and 7.7 months for ICBG. For local bone, 61.9% had bilateral fusion, 28.6% had unilateral, and 9.5% had no fusion assessed by CT. Interestingly, all patients underwent a single-level decompression alone. The volume of local bone was 14 to 22 mL for ICBG per level. Similarly, Inage et al found in 120 patients undergoing 1-, 2-, or 3-level fusion that the fusion rate with local bone graft only was 88%, 85%, and 62%, respectively, with CT scans and radiographs used. In single-level fusions, 62% fused bilaterally, and 26% fused unilaterally. The authors noted that 5 to 7 mL of bone graft per side per level was used, with no mention of decompressed levels needed to obtain that volume. Lee et al retrospectively investigated local bone grafting for single-level PLF in 182 patients with degenerative spondylolisthesis. Bilateral fusion was found in 62%, unilateral fusion in 31%, and nonunion in 7%. Follow-up was at least 18 months. Despite high fusion rates, only radiographs assessed fusion, and many patients had multilevel decompressions. Interestingly, in the discussion, they state surgical experience indicates that three levels of decompression are required to provide sufficient quantity of bone for a one-level fusion.^19^

Although the previous studies analyzed fusion rates in PLF without IB, Miura et al^13^ examined the validity of local bone as the only source of bone graft in posterior lumbar IB fusion. Local bone primarily was used to pack the cages; any remaining was placed posterolaterally. The number of levels decompressed per fusion level was not stated. Of the 32 patients, 24 patients underwent a single-level fusion and 8 underwent multilevel fusions. Fusion at 12 months was 100%, using only radiographs.

In comparison with these studies, our fusion rates are lower potentially because of various causes. In this study, stringent criteria were used to assess fusion consistent with FDA guidelines. With less stringent criteria, our fusions, particularly for multilevel decompression PLF, were closer to the published literature. Second, most of our patient cohort underwent single-level decompression with fusion, thus limiting the volume of bone graft. Third, this study was a multicenter study including many different surgeons and techniques. Fourth, the number of patients in this cohort is far more than previously published studies. Although our sample size was higher, of 310 patients in the original trial, only 231 and 182 patients at 6 months and 12 months, respectively, had complete radiographs and outcomes for review.

Ultimately, the authors believe the cause of these low fusion rates hinges on the bone graft volume. Hence, the fusion rates in the small number of patients who had a multilevel decompression demonstrated higher fusions than the single-level decompression cohort. Furthermore, the fusion rates of PLF + IB were lower than PLF despite the biomechanical advantage of this technique. The only difference hypothesized is dividing the bone graft volume to three areas of fusion rather than two areas. Interestingly, the bone graft volume was not substantially more with multilevel decompression, in particular, in patients undergoing IB fusion as the facet is completely removed at one level. We believe the reason for this discrepancy is the smaller number of patients undergoing multilevel decompression in this study. Furthermore, the quality of bone may play a more crucial role than volume. Eder et al^20^ found laminectomy bone superior in terms of cell delivery, proliferation, and mineralization than bone collected after burring. By contrast, Patel et al^21^ found in histological studies of burr shavings that viable cells without any obvious damage can be procured. However, no specific cell viability analysis was done. Nevertheless, because the fusion rates were lower with local bone only, the authors suggest that biologics may be necessary to achieve higher fusion rates when doing a one-level fusion, particularly if IB is included or doing a single-level decompression. We hypothesize with the use of biologics, fusion rates can be similar to ICBG, as various other studies have recently demonstrated.^16,22,23^

Finally, despite low fusion rates, clinical improvement was found with surgical treatment. At 12 months, the ODI improved 32 points in this study. In comparison, the multicenter SPORT study for degenerative spondylolithesis had 25.4-point improvement in the ODI at 1 year.^24^
